# Mortality Among Children Aged <5 Years Living with HIV Who Are Receiving Antiretroviral Treatment — U.S. President’s Emergency Plan for AIDS Relief, 28 Supported Countries and Regions, October 2020–September 2022

**DOI:** 10.15585/mmwr.mm7248a1

**Published:** 2023-12-01

**Authors:** Nickolas T. Agathis, Iyiola Faturiyele, Patricia Agaba, Kiva A. Fisher, Stephanie Hackett, Elfriede Agyemang, Neha Mehta, Gurpreet Kindra, Diane F. Morof, Immaculate Mutisya, Lennah Nyabiage, Katherine A. Battey, Ezeomu Olotu, Talent Maphosa, Catherine Motswere-Chirwa, Akeem T. Ketlogetswe, Jessica Mafa-Setswalo, Sikhathele Mazibuko, Maria Ines Tomo de Deus, Herminio G. Nhaguiombe, Edward M. Machage, Bridget Mugisa, Dolapo T. Ogundehin, Carolyn Mbelwa, Estella Birabwa, Monica Etima, Yakubu Adamu, Ismail Lawal, Jonah Maswai, Dorothy Njeru, Janet Mwambona, Boniface Nguhuni, Rosemary Mrina, Susan Hrapcak, George K. Siberry, Catherine Godfrey, Hilary T. Wolf

**Affiliations:** ^1^Division of Global HIV and Tuberculosis, Global Health Center, CDC; ^2^Office of HIV/AIDS, U.S. Agency for International Development, Washington, D.C.; ^3^Walter Reed Army Institute of Research, U.S. Department of Defense, Bethesda, Maryland; ^4^Henry M. Jackson Foundation for the Advancement of Military Medicine, Bethesda, Maryland; ^5^Division of Global HIV and Tuberculosis, CDC South Africa; ^6^Division of Global HIV and Tuberculosis, CDC Kenya; ^7^Division of Global HIV and Tuberculosis, CDC Namibia; ^8^Division of Global HIV and Tuberculosis, CDC Nigeria; ^9^Division of Global HIV and Tuberculosis, CDC Zimbabwe; ^10^Division of Global HIV and Tuberculosis, CDC Botswana; ^11^Ministry of Health and Wellness, Gaborone, Botswana; ^12^Division of Global HIV and Tuberculosis, CDC Eswatini; ^13^Division of Global HIV and Tuberculosis, CDC Mozambique; ^14^Division of Global HIV and Tuberculosis, CDC Tanzania; ^15^U.S. Agency for International Development, South Africa; ^16^U.S. Agency for International Development, Nigeria; ^17^U.S. Agency for International Development, Tanzania; ^18^Walter Reed Army Institute of Research, U.S. Department of Defense, Uganda; ^19^Walter Reed Army Institute of Research, U.S. Department of Defense, Nigeria; ^20^Walter Reed Army Institute of Research, U.S. Department of Defense, Kenya; ^21^Walter Reed Army Institute of Research, U.S. Department of Defense, Tanzania; ^22^Bureau of Global Health Security and Diplomacy, U.S. Department of State, Washington, D.C.

**Summary**

**What is already known about this topic?**

Globally, children aged <5 years, including children living with HIV who are not receiving antiretroviral treatment (ART), experience disproportionately high mortality.

**What is added by this report?**

Compared with older persons living with HIV receiving ART served by the U.S. President’s Emergency Plan for AIDS Relief during October 2020–September 2022, a higher proportion of children aged <5 years receiving ART died or had interrupted treatment, and a lower proportion had a suppressed HIV viral load.

**What are the implications for public health practice?**

Prioritizing and optimizing HIV and general health services for children aged <5 years living with HIV receiving ART, including those recommended in the WHO STOP AIDS Package, might help address these disproportionately poorer outcomes.

## Abstract

Globally, children aged <5 years, including those living with HIV who are not receiving antiretroviral treatment (ART), experience disproportionately high mortality. Global mortality among children living with HIV aged <5 years receiving ART is not well described. This report compares mortality and related clinical measures among infants aged <1 year and children aged 1–4 years living with HIV with those among older persons aged 5–14, 15–49, and ≥50 years living with HIV receiving ART services at all clinical sites supported by the U.S. President’s Emergency Plan for AIDS Relief. During October 2020–September 2022, an average of 11,980 infants aged <1 year and 105,510 children aged 1–4 years were receiving ART each quarter; among these infants and children receiving ART, 586 (4.9%) and 2,684 (2.5%), respectively, were reported to have died annually. These proportions of infants and children who died ranged from four to nine times higher in infants aged <1 year, and two to five times higher in children aged 1–4 years, than the proportions of older persons aged ≥5 years receiving ART. Compared with persons aged ≥5 years living with HIV, the proportions of children aged <5 years living with HIV who experienced interruptions in treatment were also higher, and the proportions who had a documented HIV viral load result or a suppressed viral load were lower. Prioritizing and optimizing HIV and general health services for children aged <5 years living with HIV receiving ART, including those recommended in the WHO STOP AIDS Package, might help address these disproportionately poorer outcomes.

## Introduction

Globally, children aged <5 years living with HIV are less likely to receive a diagnosis of HIV and be linked to antiretroviral treatment (ART) than are older persons living with HIV, and are more likely to die, especially those who are not receiving ART (*1*). Disparities in mortality and other outcomes among children compared with older persons living with HIV after initiating ART are not as well described. Given the relatively high global mortality rates among children aged <5 years in general (*2*), those living with HIV receiving ART might experience excessively high mortality compared with older persons living with HIV receiving ART. This report compares mortality and other clinical measures among infants aged <1 year and children aged 1–4 years with those among persons aged ≥5 years living with HIV receiving ART services during October 2020–September 2022, at all clinical sites supported by the U.S. President’s Emergency Plan for AIDS Relief (PEPFAR).

## Methods

PEPFAR Monitoring, Evaluation, and Reporting data collected quarterly from all PEPFAR-supported treatment sites during October 2020–September 2022 were analyzed.* Indicators included the estimated number of persons living with HIV receiving ART,^†^ mortality^§^ (i.e., reported to have died), interruption in treatment^¶^ (i.e., no clinical encounter during the 28 days after the last scheduled clinical contact), proxy viral load coverage** (i.e., documented viral load result during the previous 12 months among those assumed to be eligible for a viral load test), and viral load suppression^††^ (i.e., had a suppressed viral load result among those with a viral load result documented within the previous 12 months). Mortality was measured as the annual mean numbers and proportions of reported deaths among those receiving ART, and the other indicators are reported as quarterly mean numbers or proportions among those receiving ART^§§^; these measures were compared among children aged <5 years living with HIV receiving ART (stratified by age <1 and age 1–4 years to differentiate infants from other children aged <5 years) and older persons aged 5–14, 15–49, and ≥50 years living with HIV receiving ART. Crude mortality ratios (CMRs) were calculated comparing the proportions of reported deaths among these age groups. SAS (version 9.4; SAS Institute) was used to conduct all analyses. This activity was reviewed by CDC, deemed not research, and was conducted consistent with applicable federal law and CDC policy.^¶¶^

## Results

Among all PEPFAR-supported sites, an average of 17.9 million persons living with HIV received ART each quarter, among whom 11,980 were aged <1 year, and 105,510 were aged 1–4 years during the 2-year analysis period. Among these ART recipients, 4.9% of those aged <1 year and 2.5% of those aged 1–4 years were reported to have died annually; among older age groups these prevalences were 0.5% (5–14 years), 0.7% (15–49 years), and 1.4% (≥50 years) (Table 1) (Figure). Proportions of reported deaths among infants aged <1 year were approximately four to nine times those among older age groups: CMR = 9.2, 7.2, and 3.6 among persons living with HIV aged 5–14 years, 15–49 years, and ≥50 years, respectively. Proportions among children aged 1–4 years were approximately two to five times those among older age groups: CMR = 4.8, 3.7, and 1.9 among those aged 5–14 years, 15–49 years, and ≥50 years, respectively (Table 1). Interruptions in treatment were also more prevalent among children aged <5 years living with HIV than among persons within older age groups (<1 year and 1–4 years, 4%; 5–14 years, 2%; 15–49 years, 3%; and ≥50 years, 2%) living with HIV receiving ART; proportions were lower for proxy viral load coverage (<1 year: not reported***; 1–4 years, 66%; 5–14 years, 82%; 15–49 years, 77%; and ≥50 years, 83%), and for viral load suppression (<1 year, 78%; 1–4 years, 73%; 5–14 years, 85%; 15–49 years, 94%; and ≥50 years, 96%) (Table 2).

**TABLE 1:** Annual proportion of reported deaths and crude mortality ratios among persons living with HIV and receiving antiretroviral treatment — U.S. President’s Emergency Plan for AIDS Relief, 28 supported countries and regions,* 2021–2022^†^

Characteristic	2021			2022			2021 and 2022 (annual mean)^§^		
	% Died	No. died	No. receiving ART	% Died	No. died	No. receiving ART	% Died	No. died	No. receiving ART
**Age group, yrs**									
<1	4.4	585	13,223	5.5	587	10,737	4.9	586	11,980
1–4	2.6	2,786	108,325	2.5	2,581	102,695	2.5	2,684	105,510
5–14	0.5	2,943	537,867	0.5	2,772	534,105	0.5	2,858	535,986
15–49	0.7	94,539	13,089,351	0.6	90,672	13,984,027	0.7	92,606	13,536,689
≥50	1.4	50,001	3,488,945	1.3	51,913	3,936,499	1.4	50,957	3,712,722
**Total**	**0.9**	**150,854**	**17,237,711**	**0.8**	**148,525**	**18,568,063**	**0.8**	**149,691**	**17,902,887**
**Crude mortality ratios^¶^**									
<1 vs. 5–14	8.1	—	—	10.5	—	—	9.2	—	—
<1 vs. 15–49	6.1	—	—	8.4	—	—	7.2	—	—
<1 vs. ≥50	3.1	—	—	4.1	—	—	3.6	—	—
1–4 vs. 5–14	4.7	—	—	4.8	—	—	4.8	—	—
1–4 vs. 15–49	3.6	—	—	3.9	—	—	3.7	—	—
1–4 vs. ≥50	1.8	—	—	1.9	—	—	1.9	—	—

**Abbreviations:** ART = antiretroviral therapy; PEPFAR = U.S. President’s Emergency Plan for AIDS Relief.

* Sites from 25 PEPFAR-supported countries and three PEPFAR-supported regions were included in this analysis. The 25 countries include Angola, Botswana, Burundi, Cameroon, Côte d’Ivoire, Democratic Republic of the Congo, Dominican Republic, Eswatini, Ethiopia, Haiti, Kenya, Lesotho, Malawi, Mozambique, Namibia, Nigeria, Rwanda, South Africa, South Sudan, Tanzania, Uganda, Ukraine, Vietnam, Zambia, and Zimbabwe. The three regions include Asia Region (Burma, India, Indonesia, Kazakhstan, Kyrgyzstan, Laos, Nepal, Papua New Guinea, Philippines, Tajikistan, and Thailand), West Africa Region (Benin, Burkina Faso, Ghana, Liberia, Mali, Senegal, Sierra Leone, and Togo), and Western Hemisphere Region (Barbados, Brazil, Colombia, El Salvador, Guatemala, Guyana, Honduras, Jamaica, Nicaragua, Panama, Peru, and Trinidad and Tobago).

^†^ 2021–2022 represents fiscal years, which start in the previous October (e.g., October 2021–September 2022 represents 2022).

^§^ Proportions of reported deaths were calculated for fiscal years 2021 and 2022 by summing the reported number of deaths across the four quarters and dividing by the mean number of persons living with HIV receiving ART estimated from each of the four quarters. The number of persons living with HIV receiving ART estimated from each quarter equals the number of persons reported to be newly initiated on ART in the current quarterly reporting period plus the number reported to be receiving ART at the end of the previous quarterly reporting period.

^¶^ Crude mortality ratios are ratios of proportions of reported deaths for each age group among persons living with HIV receiving ART who are aged <1 or 1–4 years and those who are aged 5–14, 15–49, and ≥50 years.

**FIGURE:**
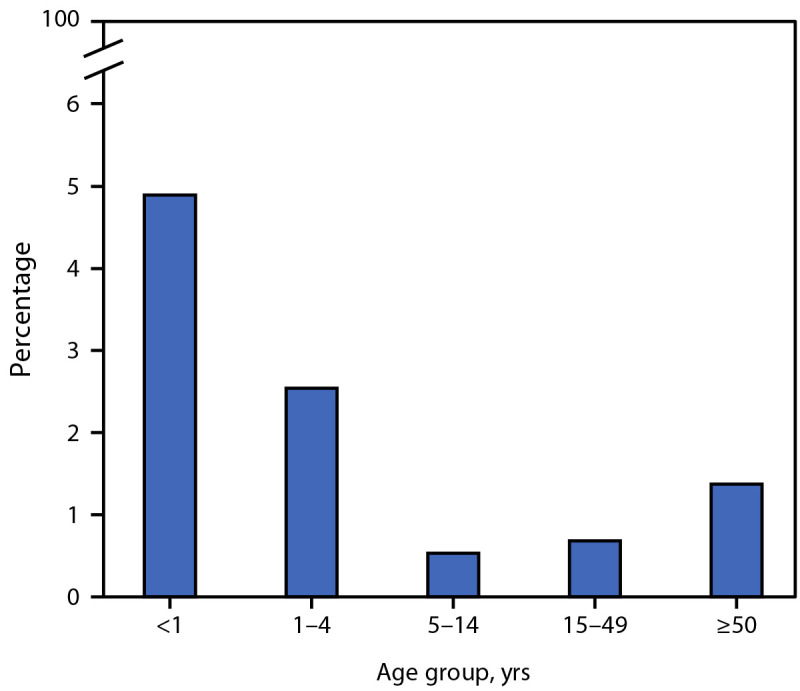
Annual percentage of reported deaths* among persons living with HIV and receiving antiretroviral treatment — U.S. President’s Emergency Plan for AIDS Relief, 28 supported countries and regions,^†^ 2021–2022^§^ **Abbreviations:** ART = antiretroviral therapy; PEPFAR = U.S. President’s Emergency Plan for AIDS Relief. * Percentage of reported deaths was calculated for fiscal years 2021 and 2022 by summing the reported number of deaths across the four quarters and dividing by the mean number of persons living with HIV receiving ART estimated from each of the four quarters. The number of persons living with HIV receiving ART estimated from each quarter equals the number of persons living with HIV reported to be newly initiated on ART in the current quarterly reporting period plus the number reported to be receiving ART at the end of the previous quarterly reporting period. ^†^ Sites from 25 PEPFAR-supported countries and three PEPFAR-supported regions were included in this analysis. The 25 countries include Angola, Botswana, Burundi, Cameroon, Côte d’Ivoire, Democratic Republic of the Congo, Dominican Republic, Eswatini, Ethiopia, Haiti, Kenya, Lesotho, Malawi, Mozambique, Namibia, Nigeria, Rwanda, South Africa, South Sudan, Tanzania, Uganda, Ukraine, Vietnam, Zambia, and Zimbabwe. The three regions include Asia Region (Burma, India, Indonesia, Kazakhstan, Kyrgyzstan, Laos, Nepal, Papua New Guinea, Philippines, Tajikistan, and Thailand), West Africa Region (Benin, Burkina Faso, Ghana, Liberia, Mali, Senegal, Sierra Leone, and Togo), and Western Hemisphere Region (Barbados, Brazil, Colombia, El Salvador, Guatemala, Guyana, Honduras, Jamaica, Nicaragua, Panama, Peru, and Trinidad and Tobago). ^§^ 2021–2022 represents fiscal years, which start in the previous October (e.g., October 2021–September 2022 represents 2022).

**TABLE 2:** Interruptions in treatment, proxy viral load coverage, and viral load suppression among persons living with HIV receiving antiretroviral treatment — U.S. President’s Emergency Plan for AIDS Relief, 28 supported countries and regions,* 2021–2022^†^^,^^§^

Characteristic	%	Numerator	Denominator
**Interruptions in treatment^¶^**			
**Age group, yrs**			
<1	4.2	509	11,980
1–4	4.0	4,224	105,510
5–14	2.4	12,735	535,986
15–49	3.0	406,565	13,536,689
≥50	2.0	74,721	3,712,722
Proxy viral load coverage**			
**Age group, yrs**			
<1**^††^**	NA	NA	NA
1–4	66.0	65,742	99,639
5–14	81.9	431,988	527,392
15–49	76.7	9,836,384	12,831,401
≥50	82.9	2,940,808	3,546,420
**Viral load suppression^§§^**			
**Age group, yrs**			
<1	78.4	2,687	3,427
1–4	73.2	48,106	65,742
5–14	85.0	367,312	431,988
15–49	94.3	9,279,763	9,836,384
≥50	96.3	2,830,747	2,940,808

**Abbreviations:** ART = antiretroviral therapy; NA = not applicable; PEPFAR = U.S. President’s Emergency Plan for AIDS Relief.

* Sites from 25 PEPFAR-supported countries and three PEPFAR-supported regions were included in this analysis. The 25 countries include Angola, Botswana, Burundi, Cameroon, Côte d’Ivoire, Democratic Republic of the Congo, Dominican Republic, Eswatini, Ethiopia, Haiti, Kenya, Lesotho, Malawi, Mozambique, Namibia, Nigeria, Rwanda, South Africa, South Sudan, Tanzania, Uganda, Ukraine, Vietnam, Zambia, and Zimbabwe. The three regions include Asia Region (Burma, India, Indonesia, Kazakhstan, Kyrgyzstan, Laos, Nepal, Papua New Guinea, Philippines, Tajikistan, and Thailand), West Africa Region (Benin, Burkina Faso, Ghana, Liberia, Mali, Senegal, Sierra Leone, and Togo), and Western Hemisphere Region (Barbados, Brazil, Colombia, El Salvador, Guatemala, Guyana, Honduras, Jamaica, Nicaragua, Panama, Peru**,** and Trinidad and Tobago).

^†^ 2021–2022 represents fiscal years which start in the previous October (e.g., October 2021–September 2022 represents 2022).

^§^ The other measures (treatment interruption, proxy viral load coverage, and viral load suppression) could not be summarized as annual estimates because, unlike mortality, treatment could have been interrupted or viral load measures received multiple times during a 1- or 2-year period. Therefore, trying to create annual estimates of these quarterly collected and reported measures would likely lead to overestimates of the actual measures because of duplication.

^¶^ Interruption in treatment is defined as not having a clinical encounter for 28 days after the last scheduled appointment or expected clinical contact. The proportions with treatment interruptions are calculated as the number of treatment interruptions in the current quarterly reporting period divided by the sum of those already receiving ART in the previous quarterly reporting period and those newly initiated on ART in the current quarterly reporting period.

** Proxy viral load coverage is defined as having a documented viral load result within the previous 12 months among those assumed to be eligible for a viral load in the current quarterly reporting period. For calculating proxy viral load coverage, the numerator is number of persons living with HIV receiving ART in the current quarterly reporting period reported to have a documented viral result during the previous 12 months, and the denominator is the number of persons receiving ART two quarters before the current quarterly reporting period. The term proxy is used because the numerator and denominator are determined from two different populations of persons.

^††^ PEPFAR Monitoring, Evaluation, and Reporting data underestimate proxy viral load coverage for infants aged <1 year living with HIV, and it has not been included in this analysis. The numerator for proxy viral load coverage in infants aged <1 year only records those with diagnoses and placed on treatment before age 6 months who received a viral load in their first year of life. The numerator excludes infants linked to ART later (aged ≥6 months) who are not eligible for a viral load until their second year of life.

^§§^ Viral load suppression is defined as having a suppressed viral load result (HIV RNA <1,000 copies/mL) among those with a documented viral load result within the previous 12 months. For calculating the proportion of persons living with HIV receiving ART with suppression, the numerator is the number of persons living with HIV receiving ART in the current quarterly reporting period with a suppressed viral load result documented within the previous 12 months, and the denominator is number of persons receiving ART in the current quarterly reporting period reported to have a documented viral result during the previous 12 months.

## Discussion

Among approximately 18 million persons living with HIV receiving ART through PEPFAR during October 2020–September 2022, prevalences of reported death were higher among children aged <5 years than among persons in older age groups, consistent with previously published findings (*3*). The additional finding that interruptions in treatment were also more common among children aged <5 years living with HIV might suggest that mortality is disproportionately underreported in this age group, because patients with interruptions in treatment or who are lost to follow-up might have died (*3*).

Among persons living with HIV receiving ART, several factors might explain the disparities in mortality among children aged <5 years compared with older persons. First, many children aged <5 years with HIV are severely immunosuppressed and at high risk for poor outcomes when they receive an HIV diagnosis and initiate ART (*4*). WHO considers all children aged <5 years living with HIV who are not clinically stable receiving ART to have advanced disease (*4*); in contrast, persons aged ≥5 years living with HIV are considered to have advanced disease only if they have a WHO stage 3 or 4 illness^†††^ or a CD4 count <200 cells/mm^3^ (*4*). Second, these findings demonstrate that children aged <5 years receiving ART have lower rates of viral load suppression along with higher rates of mortality, and viral nonsuppression is a well-described risk factor for death among children living with HIV (*5*). Finally, general mortality for persons aged <5 years, regardless of HIV status, remains high in many low-resource settings, including those where PEPFAR supports HIV programs (*2*). Factors that influence mortality in all children aged <5 years likely also influence mortality in children aged <5 years living with HIV in these settings. For example, one study from western Kenya identified common and overlapping immediate causes of death among children aged <5 years with HIV and uninfected children, including pneumonia, malnutrition, and malaria (*6*). Measures to enhance data collected at the individual level and explore causes and circumstances of death among children aged <5 years living with HIV receiving ART might help guide programs and policies aimed at preventing these deaths.

### Limitations

The findings in this report are subject to at least three limitations. First, treatment interruption and mortality estimates from PEPFAR Monitoring, Evaluation, and Reporting data are likely underreported, underestimating the actual number of deaths and interruptions in treatment among ART recipients served by PEPFAR. Second, underreporting and inconsistencies in reporting death and treatment interruption between PEPFAR-supported sites vary, which might bias or confound the findings of this analysis (i.e., site-level factors that influence completeness or consistency in reporting might also influence outcomes among children or older persons living with HIV).^§§§^ Finally, these findings might not be generalizable to children living with HIV served in non–PEPFAR-supported sites.

### Implications for Public Health Practice

Prioritizing and optimizing HIV and general health services for children aged <5 years living with HIV receiving ART might help address the disproportionately poorer outcomes they experience. Global efforts to help prevent pediatric HIV infections and optimize the entire pediatric HIV clinical cascade have intensified, including the multilateral Global Alliance to End AIDS in Children by 2030 (*7*) and the PEPFAR Accelerating Progress in Pediatrics and Prevention of Mother to Child Transmission initiative (*8*). Strategies aimed at optimizing HIV care for children living with HIV include 1) diagnosing children as early as possible, and linking them to optimized ART (especially dolutegravir-based regimens); 2) ensuring that these children continue in effective HIV care and treatment through family-centered, differentiated service delivery models (*9*); and 3) comprehensively preventing, identifying, and managing advanced HIV disease and its complications, including tuberculosis and severe acute malnutrition, according to the WHO STOP AIDS Package (*4*). Ensuring that children aged <5 years living with HIV also receive timely general pediatric services, including immunizations, micronutrient supplementation, and antimalarial treatment can also improve their health and might reduce mortality attributable to common pediatric causes of death. Enrolling children at high risk living with HIV into community-based support programs such as PEPFAR’s Orphan and Vulnerable Children program gives them access to more comprehensive services, including family-based case management and socioeconomic support, additional services that can augment the care these children receive and help them and their caregivers thrive (*8*,*10*). These strategies, as highlighted in PEPFAR’s 2023 country and regional operational planning guidance^¶¶¶^ (*8*), have the potential to prevent death, reduce the inequities experienced by children aged <5 years living with HIV, and contribute to the global measures to end AIDS among children by 2030.

## Footnotes

* Sites from 25 PEPFAR-supported countries and three PEPFAR-supported regions were included in this analysis. The 25 countries include Angola, Botswana, Burundi, Cameroon, Côte d’Ivoire, Democratic Republic of the Congo, Dominican Republic, Eswatini, Ethiopia, Haiti, Kenya, Lesotho, Malawi, Mozambique, Namibia, Nigeria, Rwanda, South Africa, South Sudan, Tanzania, Uganda, Ukraine, Vietnam, Zambia, and Zimbabwe. The three regions include Asia Region (Burma, India, Indonesia, Kazakhstan, Kyrgyzstan, Laos, Nepal, Papua New Guinea, Philippines, Tajikistan, and Thailand), West Africa Region (Benin, Burkina Faso, Ghana, Liberia, Mali, Senegal, Sierra Leone, and Togo), and Western Hemisphere Region (Barbados, Brazil, Colombia, El Salvador, Guatemala, Guyana, Honduras, Jamaica, Nicaragua, Panama, Peru, and Trinidad and Tobago).

† The estimated number of persons living with HIV receiving ART in the current quarterly reporting period equals the sum of the number of persons newly initiated on ART in the current quarterly reporting period and the number of persons receiving ART at the end of the previous quarterly reporting period.

§ Mortality is defined as having a reported death in the current quarterly reporting period.

¶ Interruption in treatment is defined as not having a clinical encounter for 28 days after the last scheduled appointment or expected clinical contact in the current quarterly reporting period. The proportions with treatment interruptions are calculated as the number of treatment interruptions in the current quarterly reporting period divided by the sum of those already receiving ART in the previous quarterly reporting period and those newly initiated on ART in the current quarterly reporting period.

** Viral load coverage is defined as having a documented viral load result within the previous 12 months among those assumed to be eligible for a viral load in the current quarterly reporting period. For calculating proxy viral load coverage, the numerator is the number of persons living with HIV receiving ART in the current quarterly reporting period reported to have a documented viral result during the previous 12 months, and the denominator is the number of persons with HIV receiving ART two quarters before the current quarterly reporting period. The term proxy is used because the numerator and denominator are determined from two distinct groups of persons living with HIV.

†† Viral load suppression is defined as having a suppressed viral load result (HIV RNA <1000 copies/mL) among those with a documented viral load result within the previous 12 months. For calculating proportion with viral load suppression, the numerator is number of persons living with HIV receiving ART in the current quarterly reporting period with a suppressed viral load result documented within the previous 12 months, and the denominator is number of persons with HIV receiving ART in the current quarterly reporting period reported to have a documented viral result during the previous 12 months.

§§ To estimate annual mortality, an annual proportion with a reported death was calculated using quarterly reported data: the numerator is the sum of reported deaths from each of the four quarters, and the denominator is the mean of the estimated number of persons living with HIV receiving ART from each of the four quarters. The other measures (treatment interruption, proxy viral load coverage, and viral load suppression) could not be summarized as annual estimates because, unlike mortality, treatment could have been interrupted or viral load measures received multiple times during a 1- or 2-year period.

¶¶ 45 C.F.R. part 46, 21 C.F.R. part 56; 42 U.S.C. Sect. 241(d); 5 U.S.C. Sect. 552a; 44 U.S.C. Sect. 3501 et seq.

*** PEPFAR Monitoring, Evaluation, and Reporting data underestimate proxy viral load coverage for children aged <1 year living with HIV, and it has not been included in this analysis. The numerator for proxy viral load coverage in infants aged <1 year only records those diagnosed and placed on treatment before age 6 months who received a viral load in their first year of life. The numerator excludes infants linked to ART later (aged ≥6 months) who are not eligible for a viral load until their second year of life.

††† Persons living with HIV are assigned a WHO-defined clinical stage between 1 and 4 based on the presence of different clinical conditions; an increase in staging likely represents a clinical worsening or progression of illness in a person living with HIV. Clinical staging criteria differ for adults and adolescents aged ≥15 years and children and adolescents aged <15 years. Notable conditions in stage 3 include pulmonary or lymph node tuberculosis, and unexplained hematologic abnormalities, fever, diarrhea, or moderate malnutrition, among other conditions. Notable conditions in stage 4 include unexplained severe malnutrition, pneumocystis jirovecii pneumonia, central nervous system complications and infections, and HIV-related malignancies, among other conditions https://iris.who.int/bitstream/handle/10665/208825/9789241549684_eng.pdf?sequence=1 (Annex 10).

§§§ Outcomes, including CMRs among persons living with HIV of different age groups, were reviewed and compared from sites that reported data on treatment loss, including treatment interruption and death, regularly (i.e., sites that reported at least one treatment loss each quarter during the 2-year period) and persons living with HIV from sites that did not report data on treatment loss regularly. The indicator that records treatment loss includes deaths, treatment interruption, transferred out, or refused or stopped treatment. CMRs were similar and substantially high in these two samples.

¶¶¶ COP/ROP 2023 guidance contains a new section of recommendations entitled “Ending Preventable Deaths in Young Children Living with HIV.”
